# Mercury spikes suggest volcanic driver of the Ordovician-Silurian mass extinction

**DOI:** 10.1038/s41598-017-05524-5

**Published:** 2017-07-13

**Authors:** Qing Gong, Xiangdong Wang, Laishi Zhao, Stephen E. Grasby, Zhong-Qiang Chen, Lei Zhang, Yang Li, Ling Cao, Zhihong Li

**Affiliations:** 1 State Key Laboratory of Geological Processes and Mineral Resources, China University of Geosciences, Wuhan, 430074 China; 2 Geological Survey of Canada, Natural Resources Canada, 3303 33rd Street N.W., Calgary, Alberta T2L 2A7 Canada; 3 State Key Laboratory of Biogeology and Environmental Geology, China University of Geosciences, Wuhan, 430074 China; 4 Wuhan Institute of Geology and Mineral Resources, Wuhan, 430223 China

## Abstract

The second largest Phanerozoic mass extinction occurred at the Ordovician-Silurian (O-S) boundary. However, unlike the other major mass extinction events, the driver for the O-S extinction remains uncertain. The abundance of mercury (Hg) and total organic carbon (TOC) of Ordovician and early Silurian marine sediments were analyzed from four sections (Huanghuachang, Chenjiahe, Wangjiawan and Dingjiapo) in the Yichang area, South China, as a test for evidence of massive volcanism associated with the O-S event. Our results indicate the Hg concentrations generally vary in parallel with TOC, and that the Hg/TOC ratios remain low and steady state through the Early and Middle Ordovician. However, Hg concentrations and the Hg/TOC ratio increased rapidly in the Late Katian, and have a second peak during the Late Hirnantian (Late Ordovician) that was temporally coincident with two main pulses of mass extinction. Hg isotope data display little to no variation associated with the Hg spikes during the extinction intervals, indicating that the observed Hg spikes are from a volcanic source. These results suggest intense volcanism occurred during the Late Ordovician, and as in other Phanerozoic extinctions, likely played an important role in the O-S event.

## Introduction

Refinements in radio-isotopic dating techniques has demonstrated a strong temporal link between Large Igneous Province (LIP) volcanism and four out of the five largest mass extinctions in the Phanerozoic, leading to suggestions that LIP events have devastating effects on global ecosystems that in some ways mirrors concerns over modern anthropogenic impacts on the planet^1–4^. The one anomaly is the Ordovician-Silurian (O-S) mass extinction, the second largest of the “Big Five” extinctions. In terms of species loss the O-S represented about 86% of marine life^5, 6^, including the elimination of graptolites^7–9^ and brachiopods^10, 11^. The triggering mechanism of the O-S mass extinction has been extensively debated, and various hypotheses have been proposed, including gamma ray burst and bolide impact^12, 13^; however, the cause still remains highly controversial. Intense Late Ordovician volcanism was also suggested as a potential extinction mechanism^14, 15^, although no clear geologic record of a Late Ordovician LIP event has been demonstrated. The age of the event makes it possible that any geologic record has been lost^15^. Given this, we tested for indirect evidence of volcanism at the O-S extinction boundary by examining the Hg record from classic sections in south China. Here we show that significant spikes in Hg concentrations occur at the O-S extinction, along with Hg stable isotope data that indicate a volcanic source for these spikes. We suggest that this reflects Hg loading by a LIP event. If correct, these results demonstrate that LIP events have indeed played a critical role in the evolution of life through time.

## Mercury as a Proxy for LIP events

Volcanic eruptions are the main natural source of Hg to the environment^16^. Sanei *et al*.^17^ demonstrated that the Siberian Trap eruption, the largest LIP in the geologic record and also associated with the largest Phanerozoic extinction event, left a characteristic mercury spike as a fingerprint in the rock record. Subsequent work has confirmed Hg spikes associated with other LIP events associated with mass extinction intervals^18–23^. Grasby *et al*.^21^ also demonstrated that background Hg levels appear constant over geologic time, showing that Hg spikes associated with LIPs are truly anomalous features of the rock record. Organic matter (OM) has a strong capacity for absorbing dissolved Hg in marine conditions^21^, whereby Hg deposited in the ocean through wet and dry fall is mainly captured by OM and sequestered into sediments through geologic time. Excess mercury flux to the environment during periods of major volcanic eruption can overwhelm this natural system, creating a mercury spike relative to total organic carbon (TOC) in the rock record^17^, as a key indicator of massive volcanism.

Hg stable isotope data can further verify a volcanic source of Hg spikes^20, 24^. According to Blum *et al*.^25^, Hg stable isotopes exhibit both mass-dependent fractionation (MDF, reported as δ^202^Hg) and mass-independent fractionations (MIF, reported as Δ^199^Hg) in the environment and can be used as multi-dimensional tracers to discriminate Hg sources, transport and cycling^25–28^. We used the nomenclature of Blum and Bergquist^29^ to describe mercury isotope compositions, whereby Hg isotopic composition is reported in δ^202^Hg notation in units of permil (‰) referenced to the NIST SRM 3133 Hg standard:

1 $${\rm{\delta }}{}^{202}{\rm{H}}{\rm{g}}(\textperthousand )=[({}^{202}{\rm{H}}{\rm{g}}/{}^{198}{\rm{H}}{{\rm{g}}}_{{\rm{sample}}})/({}^{202}{\rm{H}}{\rm{g}}/{}^{198}{\rm{H}}{{\rm{g}}}_{{\rm{standard}}})-1]\times 1000$$

MIF of Hg isotopes is expressed in Δ notation (Δ^xxx^Hg) in units of permil (‰), describing the difference between the measured δ^xxx^Hg and the theoretically predicted δ^xxx^Hg value:

2 $${}^{{\rm{xxx}}}{\rm{H}}{\rm{g}}(\textperthousand )\approx {\rm{\delta }}{}^{{\rm{xxx}}}{\rm{H}}{\rm{g}}-{\rm{\delta }}{}^{202}{\rm{H}}{\rm{g}}\times {\rm{\beta }}$$

where β is equal to 0.2520 for ^199^Hg, 0.5024 for ^200^Hg, and 0.7520 for ^201^Hg.

## Ordovician record in South China

**Figure 1:**
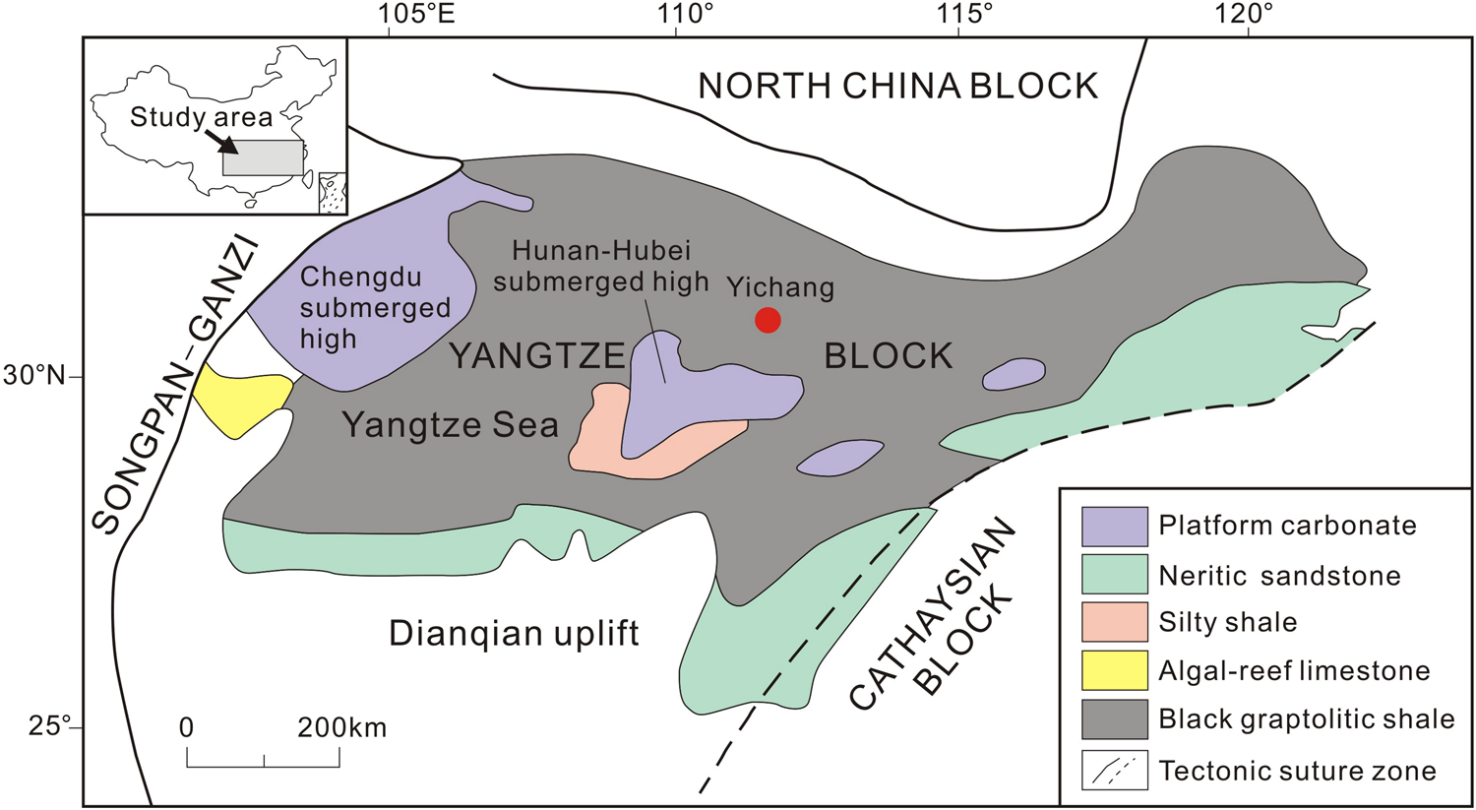
Palaeogeographic map of Yangtze block area during the Late Ordovician. Modified from Zhou *et al*.^34^. The study sections are at Yichang, South China.

We investigated the geochemistry of Hg in four sections of Ordovician and early Silurian strata, namely Huanghuachang, Chenjiahe, Wangjiawan and Dingjiapo, all within the Yichang area of South China (Fig. 1). Regarded as the classic locality of the Ordovician, the continuous and fossiliferous Ordovician strata are completely preserved in this area^30, 31^ that includes two GSSPs (Early-Middle Ordovician boundary at Huanghuachang section, and the Ordovician–Silurian boundary at the Wangjiawan section)^32^. During the Early-Middle Ordovician, the Yangtze Platform was covered by an extensive epeiric sea and was dominated by extensive shallow-marine carbonates with abundant conodonts fossils^33^. The strata of the Late Ordovician to Early Silurian interval represented the deep water environment in the Yangtze platform, making these ideal environments to examine Hg loading to the marine system.

The composite of the previous three sections provides a nearly complete Ordovician succession. In ascending order, the Huanghuachang section includes Hunghuayuan, Dawan, Kuniutan, Miaopo, and Pagoda formations. The Chenjiahe section includes the Linhsiang Formation. The Wangjiawan section records the O-S extinction internal through the Wufeng, Kuanyinchiao, and Lungmachi formations. For comparative purposes, we also examined the Dingjiapo section in the Yichang area, which also records the O-S extinction. At Dingjiapo, the Wufeng, and Lungmachi formations are both characterized by black shale that contains rich graptolite fossils^35–37^, representing deep water conditions, while the Kuanyinchiao Formation is dominated by argillaceous limestone with Hirnantian fauna fossils that record a drop in eustatic sea level^38^ and lowstand conditions during the Hirnantian glacial maximum^10, 34, 38^.

The O-S boundary at the Wangjiawan section, GSSP of the base of Hirnantian Stage (Latest Ordovician), was marked by two extinction phases^39–42^. The first lasted from the Late Katian (*D. mirus* Zone) to the Early Hirnantian (*N. extraordinarius* Zone), and coincided with the development of Gondwanan glaciation^42, 43^. At the beginning of Hirnantian Stage, the climate dramatically changed from warm to cold, and a large-scale drawdown of eustatic sea-level provided oxic conditions in the oceans, corresponding with the positive excursion in δ^13^C values of both carbonate and organic carbon^40^. The marine organisms that had been adapted to warm waters suffered massive demise^34, 40^, while the Hirnantian fauna accustomed to cold and shallow waters became prosperous and widespread all over the world^7, 8^. The second extinction pulse occurred at the beginning of Late Hirnantian (from *N. extraordinarius* Zone to *N. persculptus* Zone)^42^. It began with the return of globally warm weather, followed by the melt of Gondwanan ice sheet and rapid rise in eustatic sea-level, leading to severe anoxic conditions reported by a large negative shift in δ^13^C values. Those organisms, just like the Hirnantian fauna that adapted to shallow and cold water environments, had great declines^40^. The lithologies of sediments in Wangjiawan section, as the black shale in Wufeng and Lungmachi Formations and the argillaceous limestone in Kuanyinchiao Formation, distinctly record the remarkable swings from anoxic to oxic and back to anoxic environments through the O-S boundary.

## Mercury Spikes at the O-S extinction

**Figure 2:**
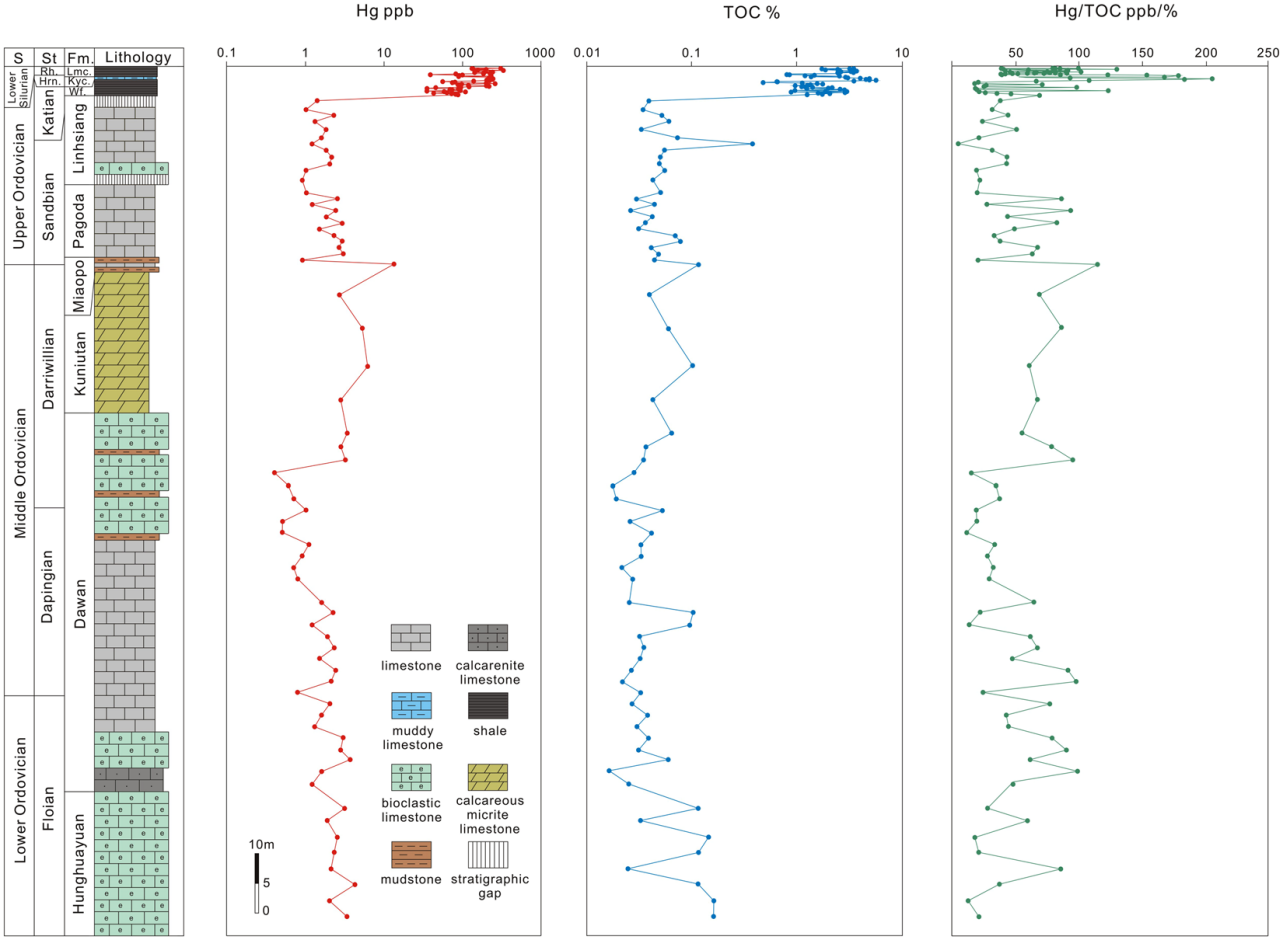
Geochemical plots of trends in Hg, TOC (total organic carbon) and Hg/TOC for Yichang sections through Ordovician and Lower Silurian. Yichang sections include Huanghuachang, Chenjiahe and Wangjiawan. Abbreviation: S = Series, St = Stage, Fm. = Formation, Wf. = Wufeng, Kyc. = Kuanyinchiao, Lmc = Lungmachi, Hrn. = Hirnantian, Rh. = Rhuddanian.

Hg concentrations are low throughout the Early and Middle Ordovician (generally <5 ppb) (Fig. 2). However, in Late Ordovician and Early Silurian time, the Hg concentrations increased rapidly to 200 ppb in the upper Katian, and then show a second prominent Hg spike (>300 ppb) at the Ordovician-Silurian (O-S) boundary.

**Figure 3:**
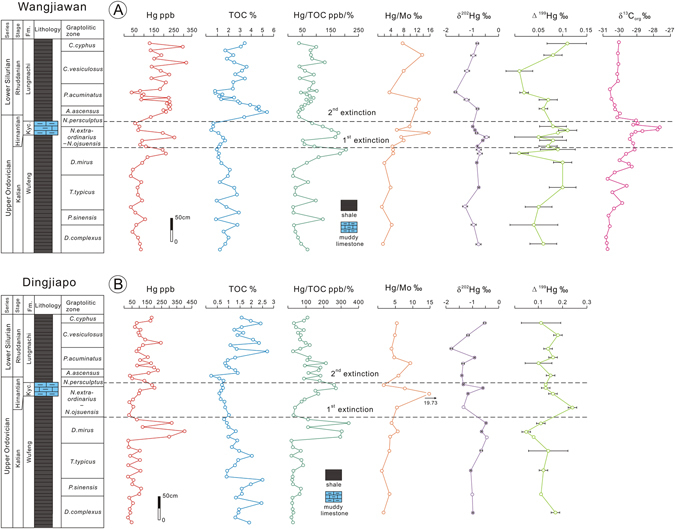
Geochemical plots for Wangjiawan (**A**) and Dingjiapo (**B**) sections across the Ordovician-Silurian boundary. Plots show variation of δ^13^C_org_ for Wangjiawan and Hg, TOC, Hg/TOC, Hg/Mo, δ^202^Hg and Δ^199^Hg for Wangjiawan and Dingjiapo. Graptolitic zone is from Chen *et al*.^35^ and δ^13^C_org_ data are from Yan *et al*.^40^. Abbreviation: Fm. = Formation, Kyc. = Kuanyinchiao.

Total organic carbon (TOC) values ranged within a low level from 0.02 to 0.1% during the Early and Middle Ordovician. In contrast, high TOC values, ranging from 1 to 5%, are recorded in the Late Ordovician and Early Silurian. In general TOC values varied consistently with Hg concentrations, while the balance between them broke down near the O-S boundary as showed by the elevated Hg/TOC ratio. The Hg/TOC ratio fluctuated at a relatively low level of ~100 throughout the Ordovician and Early Silurian. However, there is a peak of 200 in the Wufeng Formation in the Late Katian *D. mirus* Zone, followed by a drop to 60 in the lower *N. extraordinarius–N. ojsuensis* Zone. Subsequently, a second peak of 180 occurred in the Kuanyinchiao Formation of the Early Hirnantian Stage, in the upper *N. extraordinarius–N. ojsuensis* Zone (these peaks are shown in greater detail in Fig. 3).

Hg and TOC data from the Dingjiapo section are also shown in Fig. 3. Here Hg varied from 20 to 200 ppb, with an anomalous value of 400 ppb in the upper Katian (higher than the Hg peak in the Wangjiawan section). The TOC contents, typically less that 1%, also showed a spike of 2% in Late Katian, followed by a drop to about 0.7% and then return to ~2% in the upper Hirnantian. The Hg/TOC ratio remained relatively stable, around 50, in middle Katian and then increased sharply upwards reaching a peak of 350 at the upper Katian *D.mirus* Zone, followed by a shift to ~40, and gradual increase to 260 as a second peak in the uppermost *N. extraordinarius–N. ojsuensis* Zone. The two positive peaks of Hg/TOC ratios at Dingjiapo are consistent with those at Wangjiawan, clearly demonstrating that a double mercury anomaly at the O-S boundary is reproducible at different sections.

The Hg/Mo ratios ranged from 1.24 to 15.67‰ in Wangjiawan and 1.14 to 19.73‰ in Dingjiapo. In both sections, the Hg/Mo ratios showed small increases associated with the Hg/TOC spikes for the first pulse of extinction, and then sharply rise to their largest positive peaks for the second (Fig. 3). The Hg isotope data from the two O-S boundary sections of Wangjiawan and Dingjiapo are shown in Fig. 3. All the δ^202^Hg values are negative and range from −1.62 to −0.45‰ at Wangjiawan and −1.62 to −0.40‰ at Dingjiapo, and both sections show negligible but consistent negative deviations in δ^202^Hg across the extinction. Furthermore, Wangjiawan shows positive Δ^199^Hg values (~0.08 to ~0.11‰), whereas Δ^199^Hg values in Dingjiapo display a higher spike of 0.16 to 0.24‰ during the two phases of extinction, compared with the pre-extinction and post-extinction samples. However, little to no variation in Hg isotope values is observed associated with the large Hg spikes at the extinction boundary.

## A Late Ordovician LIP event

Our data show a covariant relationship between Hg and organic matter (OM) throughout the Ordovician, with the exception of two significant Hg spikes near the O-S boundary. The first one is in the Late Katian *D. mirus* Zone, coincident with the first (Late Katian) extinction episode. The second Hg spike occurs in the Kuanyinchiao Formation in the Early Hirnantian Stage, upper *N. extraordinarius*–*N. ojsuensis* Zone, simultaneous with the second (Late Hirnantian) extinction pulse. Anoxia events can be partly linked to the LIP record^44^, and this can increase TOC that then increases Hg. However, the spikes in Hg/TOC ratios indicate excess Hg above what might be related to increased TOC alone. Likewise, the Hg/Mo ratio shows a small increase related to the Hg/TOC spikes for the first pulse of extinction and a larger one for the second. As Mo is a typical redox sensitive element which increases in anoxia conditions, the Hg/Mo ratios increase associated with the Hg/TOC spikes demonstrates that Hg acts independent of Mo and other redox sensitive elements, implying increased Hg flux to the ocean basin at those times rather than redox driven increased Hg drawdown.

The Hg isotopic compositions help to trace the origin and pathways of these Hg spikes. Mercury can undergo complex geochemical transformation processes in the environment, that may induce mass dependent fractionation (MDF) and mass independent fractionation (MIF) of Hg isotopes^25, 45, 46^. MDF can occur during various physical, chemical, or biological transformations of Hg, whereas MIF is generated through a more limited set of pathways (mostly photochemical), making it a more conservative tracer^46^. Generally, Hg derived from volcanic eruptions or geogenic sources has insignificant MIF (Δ^199^Hg ~ 0‰) with δ^202^Hg values approximately between −2.0‰ and 0‰ (e.g., Zambardi *et al*.)^47^. When released to the environment, Hg cycles through the atmospheric, marine, and terrestrial reservoirs^46^. In the atmosphere, odd-isotope MIF can be generated through the aqueous photoreduction of Hg^2+^ ^48^, such as in surface waters and cloud droplets, resulting in excess odd-isotope (i.e., positive Δ^199^Hg values) in the residual aqueous Hg^2+^ pool. Thus, atmospheric Hg^2+^, and sediments dominated by atmospheric Hg^2+^ deposition, tend to have positive Δ^199^Hg values (e.g. Thibodeau *et al*.^46^, Gehrke *et al*.^49^). Grasby *et al*.^24^ proposed that the background Δ^199^Hg values reflect a dominant volcanic source of Hg by atmospheric Hg^2+^ deposition and/or enhanced Hg^2+^ photoreduction in the water column. In our study, the Wangjiawan and Dingjiapo sections had limited variability in Δ^199^Hg. Shifts in Δ^199^Hg values at two extinction levels are negligible, suggesting that the Hg spikes are not related to changes in the background Hg source, but rather just enhanced loading from those sources (volcanoes). The overall positive shifts of Δ^199^Hg values probably suggest that the spikes of Hg are related to the Hg^2+^ absorbed from the atmosphere by volcanic plume particles, instead of direct atmospheric deposition from volcanoes themselves. This finding is consistent with the stable Hg values of Buchanan Lake at the latest Permian extinction (LPE), which also reflected a dominant volcanic source for an Hg spike^24^.

Previous work has shown that catastrophic volcanism releases huge volumes of Hg into the atmosphere, dominantly from volcanic emissions and also in some cases a minor additional component from organic combustion when lavas intrude through sedimentary basins^22, 50^. Subsequent Hg deposition from air into marine environments can overwhelm the normal organic buffering of Hg, leading to spikes in the Hg/TOC ratio in the rock record^17, 21^. As such, we argue that the two Hg spikes we observe, that are temporally coincident with the two main O-S extinction pulses, represent enhanced Hg loading to marine environments due to greatly increased volcanic activity. This interpretation is supported by our Hg stable isotope data.

There are no defined LIP events in the geologic record coincident with the O-S extinction, however they may not have been preserved and/or refined age dates of candidate volcanics have not yet been obtained. Recently Ernst *et al*.^44^ suggested candidate LIP events do occur at this time, most notably the Suordakh intraplate event in eastern Siberia which is ca. 440 Ma^51^, the mafic events of Ongnyeobong Formation volcanics in South Korea^52^ and flood basalts in Argentina, southern South America^53^ that are also of approximately the right age, but without sufficiently precise age dates to confirm a direct link.

## Conclusions

The analyses of Hg and TOC values from sections in the Yangtze Platform show consistent background values and Hg/TOC ratios through the Early and Middle Ordovician. However, distinct spikes in both Hg concentrations and Hg/TOC occur in the Late Katian and Late Hirnantian, coincident with the two main extinction pulses across the O-S boundary. This result was reproduced in two locations (Wangjiawan and Dingjiapo). Hg stable isotope data show little to no MIF signatures associated with the large Hg spikes within the extinction interval, providing direct evidence that these Hg spike are indeed from a volcanic source. Therefore, we conclude that similar to other Phanerozoic mass extinctions, major volcanic eruptions may have triggered the O-S mass extinction events. If correct, it appears that LIPs have planned a major role in the evolution of life throughout Earth history. This is likely through the broad global impacts that release of volcanic gases would have had on the atmosphere, terrestrial environments, and marine systems. Such eruptions are characterized by significant release of toxic metals, ozone depleting chemicals, greenhouse gases, and induced ocean acidification and anoxia^4, 44^ many of the same concerns related to modern impacts of industrialization. More work on the deep time record of LIP events could provide invaluable information on major evolutionary events in Earth history.

## Methods

In the field, samples from Ordovician and early Silurian rocks were collected at centimeter intervals. Samples were collected from cleaned fresh exposed outcrop. Analytical methods were based on those presented by Grasby *et al*.^21^. In the laboratory, samples were chipped into pieces and selected to remove weathered surfaces before powdering. Clean samples were powdered in a Retsch® MM400 oscillating mill and dried at 60 °C to constant weight. For the total organic carbon (TOC) analysis, the powered samples were treated by hydrochloric acid to remove the inorganic carbon. The TOC content was measured by a Elementar® vario Macro cube. Total mercury concentrations (THg) were measured by a LECO® AMA254 mercury analyzer. Analyses of Hg and TOC were performed at State Key Laboratory of Geological Processes and Mineral Resources, China University of Geosciences, Wuhan. The complete data set of Hg and TOC values and lithostratigraphic information are listed in Supplementary Tables S1 and S2.

The concentrations of trace elements, including Mo, were determined using an Agilent® 7500a ICP-MS at State Key Laboratory of Geological Processes and Mineral Resources, China University of Geosciences, Wuhan. The Mo data are listed in Supplementary Tables S1 and S2.

Hg isotopes of Ordovician-Silurian transition from Wangjiawan and Dingjiapo sections were analysed by methods described previously (see Yin *et al*.^54^, Yin *et al*.^55^ and references therein). Approximately 0.5 to 1 g of homogenized samples were digested with a 12 mL aqua regia (HCl:HNO_3_ = 3:1, v-v) and heated in water at 95 °C for 3 hours. After centrifugation, the precipitates were removed, and the clear supernatants extract were diluted by adding Milli-Q water to Hg concentrations of ~2 ng/mL in solutions with acid concentration of <20%. An internal Tl standard (NIST SRM 997) was used to correct instrument mass bias. NIST SRM 3133 was prepared as Hg bracketing standard to match matrix and Hg concentrations. The concentrations of Hg and acid in the bracketing NIST SRM 3133 and sample solutions were matched within 10% to reduce the matrix dependent mass bias. We measured NIST SRM 3133 solutions once every 3 samples. Concentrations of Hg and acid matrices of samples were matched to the neighbor measured NIST SRM 3133 solutions. We also measured NIST SRM 3177 once every 10 samples to examine the instrument accuracy and calculate measuring error of samples. Hg isotopic ratios were determined by a Nu-Plasma multi-collector inductively coupled plasma mass spectrometer (MC-ICP-MS) at the State Key Laboratory of Environmental Geochemistry, Institute of Geochemistry, Chinese Academy of Sciences, Guiyang, China. The Hg isotopes data of Wangjiawan and Dingjiapo can be found in Supplementary Tables S1 and S2.

## Electronic supplementary material

Supplementary Tables
