# Supplementary material for: Mercury spikes suggest volcanic driver of the Ordovician-Silurian mass extinction
Source: Sci Rep. 2017 Jul 13;7:5304. doi: 10.1038/s41598-017-05524-5 (PMC5509715; doi:10.1038/s41598-017-05524-5)
Supplement: Supplementary file 1 — Supplementary Tables [file 41598_2017_5524_MOESM1_ESM.doc]

Mercury spikes suggest volcanic driver of the Ordovician-Silurian mass extinction

Qing Gong^1^, Xiangdong Wang^1^, Laishi Zhao^1^*, Stephen E. Grasby^2^*, Zhong-Qiang Chen^1,3^, Lei Zhang^1^, Yang Li^1^, Ling Cao^1^ & Zhihong Li^4^

^1^ State Key Laboratory of Geological Processes and Mineral Resources, China University of Geosciences, Wuhan 430074, China

^2^ Geological Survey of Canada, Natural Resources Canada, 3303 33rd Street N.W., Calgary, Alberta T2L 2A7, Canada

^3^ State Key Laboratory of Biogeology and Environmental Geology, China University of Geosciences, Wuhan 430074, China

^4^ Wuhan Institute of Geology and Mineral Resources, Wuhan 430223, China

*Corresponding author: Laishi Zhao (zlscug@163.com); Steve Grasby ([steve.grasby@canada.ca](mailto:steve.grasby@canada.ca)).

**Contents**

**Supplementary Table S1:** Hg concentrations, TOC values, Mo values and Hg isotopic compsition for Yichang samples. These data are plotted in Figure 2 and Figure 3 and were used to select a representative subset of samples for Hg isotope analyses in this study.

**Supplementary Table S2:** Hg concentrations, TOC values, Mo values and Hg isotopic compsition for Dingjiapo samples. These data are plotted in Figure 3 and were used to select a representative subset of samples for Hg isotope analyses in this study.

**Supplementary Table S1.** Hg concentrations, TOC values, Mo values and Hg isotopic compsition for Yichang samples. These data are plotted in Figure 2 and Figure 3 and were used to select a representative subset of samples for Hg isotope analyses in this study.

| Sample No. | Formation | Height (m) | Hg (ppb) | TOC (%) | Hg/TOC (ppb/%) | Mo (ppm) | Hg/Mo (‰) | δ^202^Hg (‰) | 2σ | Δ^199^Hg (‰) | 2σ |
| --- | --- | --- | --- | --- | --- | --- | --- | --- | --- | --- | --- |
| WJW-52 | Lungmachi | 147.16 | 131.8 | 3.39 | 38.91 | 17.11 | 7.70 | -0.79 | 0.06 | 0.11 | 0.04 |
| WJW-51 | Lungmachi | 147.10 | 302.5 | 3.04 | 99.48 |  |  |  |  |  |  |
| WJW-50 | Lungmachi | 147.04 | 136.3 | 1.70 | 80.13 |  |  |  |  |  |  |
| WJW-49 | Lungmachi | 146.98 | 192.1 | 2.38 | 80.67 | 14.06 | 13.67 | -0.95 | 0.14 | 0.08 | 0.02 |
| WJW-48 | Lungmachi | 146.92 | 155.2 | 1.83 | 85.01 |  |  |  |  |  |  |
| WJW-47 | Lungmachi | 146.86 | 320.5 | 2.47 | 129.96 |  |  |  |  |  |  |
| WJW-46 | Lungmachi | 146.80 | 143.3 | 3.55 | 40.41 |  |  |  |  |  |  |
| WJW-45 | Lungmachi | 146.74 | 189.1 | 3.15 | 60.01 | 23.63 | 8.00 | -1.19 | 0.12 | 0.01 | 0.03 |
| WJW-44 | Lungmachi | 146.68 | 233.2 | 2.56 | 91.20 |  |  |  |  |  |  |
| WJW-43 | Lungmachi | 146.62 | 141.1 | 3.39 | 41.62 |  |  |  |  |  |  |
| WJW-42 | Lungmachi | 146.56 | 216.0 | 2.39 | 90.43 |  |  |  |  |  |  |
| WJW-41 | Lungmachi | 146.50 | 180.9 | 2.38 | 75.97 |  |  |  |  |  |  |
| WJW-40 | Lungmachi | 146.44 | 81.0 | 0.80 | 101.81 |  |  |  |  |  |  |
| WJW-39 | Lungmachi | 146.38 | 38.3 | 0.83 | 46.31 | 10.34 | 3.70 | -1.62 | 0.08 | 0.02 | 0.01 |
| WJW-38 | Lungmachi | 146.32 | 96.5 | 1.45 | 66.46 |  |  |  |  |  |  |
| WJW-37 | Lungmachi | 146.26 | 89.5 | 1.16 | 77.01 |  |  |  |  |  |  |
| WJW-36 | Lungmachi | 146.20 | 223.9 | 2.48 | 90.14 |  |  |  |  |  |  |
| WJW-35 | Lungmachi | 146.14 | 87.1 | 1.34 | 65.08 | 6.89 | 12.64 | -1.20 | 0.15 | 0.07 | 0.02 |
| WJW-34 | Lungmachi | 146.08 | 229.2 | 2.82 | 81.24 |  |  |  |  |  |  |
| WJW-33 | Lungmachi | 146.02 | 216.4 | 2.98 | 72.52 |  |  |  |  |  |  |
| WJW-32 | Lungmachi | 145.96 | 231.7 | 3.91 | 59.26 |  |  |  |  |  |  |
| WJW-31 | Lungmachi | 145.90 | 224.5 | 4.67 | 48.12 |  |  |  |  |  |  |
| WJW-30 | Lungmachi | 145.84 | 231.3 | 4.46 | 51.89 | 19.75 | 11.71 | -0.79 | 0.08 | 0.06 | 0.01 |
| WJW-29 | Lungmachi | 145.78 | 198.3 | 4.72 | 42.00 |  |  |  |  |  |  |
| WJW-28 | Lungmachi | 145.72 | 210.8 | 5.39 | 39.11 |  |  |  |  |  |  |
| WJW-27 | Lungmachi | 145.66 | 133.8 | 3.38 | 39.58 |  |  |  |  |  |  |
| WJW-26 | Kuanyinchiao | 145.60 | 54.9 | 0.63 | 86.77 |  |  |  |  |  |  |
| WJW-25 | Kuanyinchiao | 145.52 | 76.8 | 0.63 | 122.88 | 7.83 | 9.81 | -0.97 | 0.02 | 0.08 | 0.03 |
| WJW-24 | Kuanyinchiao | 145.44 | 71.8 | 0.47 | 153.39 | 12.38 | 5.80 | -0.89 | 0.02 | 0.11 | 0.02 |
| WJW-23 | Kuanyinchiao | 145.36 | 195.3 | 1.09 | 178.60 | 12.46 | 15.67 | -0.83 | 0.08 | 0.09 | 0.00 |
| WJW-22 | Wufeng | 145.28 | 253.5 | 1.51 | 168.18 | 35.15 | 7.21 | -0.45 | 0.12 | 0.05 | 0.05 |
| WJW-21 | Wufeng | 145.20 | 96.2 | 1.70 | 56.75 | 12.38 | 7.77 | -0.60 | 0.02 | 0.08 | 0.03 |
| WJW-20 | Wufeng | 145.12 | 85.0 | 1.32 | 64.56 |  |  |  |  |  |  |
| WJW-19 | Wufeng | 145.04 | 115.7 | 1.23 | 94.01 | 25.09 | 4.61 | -0.80 | 0.01 | 0.07 | 0.02 |
| WJW-18 | Wufeng | 144.96 | 194.2 | 0.95 | 205.42 | 40.62 | 4.78 | -0.74 | 0.13 | 0.09 | 0.04 |
| WJW-17 | Wufeng | 144.88 | 210.4 | 1.15 | 183.48 | 44.01 | 4.78 | -0.70 | 0.14 | 0.01 | 0.02 |
| WJW-16 | Wufeng | 144.80 | 118.5 | 1.09 | 108.50 |  |  |  |  |  |  |
| WJW-15 | Wufeng | 144.68 | 91.9 | 1.37 | 67.23 | 47.49 | 1.94 | -0.82 | 0.02 | 0.10 | 0.02 |
| WJW-14 | Wufeng | 144.56 | 44.5 | 2.09 | 21.25 |  |  |  |  |  |  |
| WJW-13 | Wufeng | 144.44 | 34.7 | 1.94 | 17.92 |  |  |  |  |  |  |
| WJW-12 | Wufeng | 144.32 | 86.6 | 1.21 | 71.69 |  |  |  |  |  |  |
| WJW-11 | Wufeng | 144.20 | 68.2 | 2.50 | 27.33 | 18.14 | 3.76 | -0.74 | 0.02 | 0.10 | 0.03 |
| WJW-10 | Wufeng | 144.08 | 69.9 | 2.74 | 25.50 |  |  |  |  |  |  |
| WJW-9 | Wufeng | 143.96 | 91.6 | 0.93 | 98.29 |  |  |  |  |  |  |
| WJW-8 | Wufeng | 143.84 | 34.4 | 1.80 | 19.07 | 27.79 | 1.24 | -1.26 | 0.12 | 0.05 | 0.03 |
| WJW-7 | Wufeng | 143.72 | 57.0 | 2.89 | 19.73 |  |  |  |  |  |  |
| WJW-6 | Wufeng | 143.60 | 106.2 | 0.86 | 123.21 |  |  |  |  |  |  |
| WJW-5 | Wufeng | 143.48 | 61.5 | 2.77 | 22.18 | 14.58 | 4.22 | -0.94 | 0.13 | 0.04 | 0.05 |
| WJW-4 | Wufeng | 143.35 | 42.1 | 1.55 | 27.24 |  |  |  |  |  |  |
| WJW-3 | Wufeng | 143.24 | 71.2 | 1.98 | 36.03 |  |  |  |  |  |  |
| WJW-2 | Wufeng | 143.12 | 80.1 | 1.70 | 47.08 | 52.11 | 1.54 | -0.75 | 0.11 | 0.06 | 0.03 |
| WJW-1 | Wufeng | 143.00 | 84.9 | 1.23 | 69.30 |  |  |  |  |  |  |
| CJH-12 | Linhsiang | 141.50 | 1.4 | 0.04 | 36.55 |  |  |  |  |  |  |
| CJH-11 | Linhsiang | 140.00 | 1.0 | 0.03 | 29.67 |  |  |  |  |  |  |
| CJH-10 | Linhsiang | 138.80 | 2.3 | 0.05 | 45.01 |  |  |  |  |  |  |
| CJH-9 | Linhsiang | 137.60 | 1.3 | 0.06 | 21.81 |  |  |  |  |  |  |
| CJH-8 | Linhsiang | 136.40 | 1.8 | 0.03 | 54.88 |  |  |  |  |  |  |
| CJH-7 | Linhsiang | 135.20 | 1.6 | 0.07 | 22.41 |  |  |  |  |  |  |
| CJH-6 | Linhsiang | 134.00 | 1.2 | 0.37 | 3.23 |  |  |  |  |  |  |
| CJH-5 | Linhsiang | 132.80 | 1.8 | 0.05 | 32.91 |  |  |  |  |  |  |
| CJH-4 | Linhsiang | 131.60 | 2.1 | 0.05 | 42.42 |  |  |  |  |  |  |
| CJH-3 | Linhsiang | 130.40 | 2.0 | 0.05 | 41.32 |  |  |  |  |  |  |
| CJH-2 | Linhsiang | 129.20 | 1.0 | 0.05 | 18.52 |  |  |  |  |  |  |
| CJH-1 | Linhsiang | 128.00 | 0.9 | 0.04 | 21.53 |  |  |  |  |  |  |
| HHC-55 | Pagoda | 125.50 | 1.0 | 0.05 | 20.16 |  |  |  |  |  |  |
| HHC-54 | Pagoda | 124.50 | 2.5 | 0.03 | 86.21 |  |  |  |  |  |  |
| HHC-53 | Pagoda | 123.50 | 1.2 | 0.04 | 27.91 |  |  |  |  |  |  |
| HHC-52 | Pagoda | 122.50 | 2.4 | 0.03 | 93.39 |  |  |  |  |  |  |
| HHC-51 | Pagoda | 121.50 | 1.8 | 0.04 | 43.58 |  |  |  |  |  |  |
| HHC-50 | Pagoda | 120.50 | 2.9 | 0.04 | 81.92 |  |  |  |  |  |  |
| HHC-49 | Pagoda | 119.50 | 1.5 | 0.03 | 48.86 |  |  |  |  |  |  |
| HHC-48 | Pagoda | 118.50 | 2.3 | 0.07 | 33.43 |  |  |  |  |  |  |
| HHC-47 | Pagoda | 117.50 | 2.9 | 0.08 | 38.06 |  |  |  |  |  |  |
| HHC-46 | Pagoda | 116.50 | 2.7 | 0.04 | 67.00 |  |  |  |  |  |  |
| HHC-45 | Pagoda | 115.50 | 3.0 | 0.05 | 63.16 |  |  |  |  |  |  |
| HHC-44 | Miaopo | 114.50 | 0.9 | 0.04 | 20.69 |  |  |  |  |  |  |
| HHC-43 | Miaopo | 113.00 | 13.2 | 0.11 | 115.08 |  |  |  |  |  |  |
| HHC-42 | Kuniutan | 107.00 | 2.7 | 0.04 | 68.18 |  |  |  |  |  |  |
| HHC-41 | Kuniutan | 102.00 | 5.2 | 0.06 | 86.24 |  |  |  |  |  |  |
| HHC-40 | Kuniutan | 97.00 | 6.1 | 0.10 | 60.82 |  |  |  |  |  |  |
| HHC-39 | Kuniutan | 92.00 | 2.8 | 0.04 | 66.83 |  |  |  |  |  |  |
| HHC-38 | Dawan | 87.00 | 3.4 | 0.06 | 55.02 |  |  |  |  |  |  |
| HHC-37 | Dawan | 85.00 | 2.8 | 0.04 | 79.10 |  |  |  |  |  |  |
| HHC-36 | Dawan | 83.00 | 3.2 | 0.03 | 94.96 |  |  |  |  |  |  |
| HHC-35 | Dawan | 81.00 | 0.4 | 0.03 | 14.81 |  |  |  |  |  |  |
| HHC-34 | Dawan | 79.00 | 0.6 | 0.02 | 34.68 |  |  |  |  |  |  |
| HHC-33 | Dawan | 77.00 | 0.7 | 0.02 | 38.04 |  |  |  |  |  |  |
| HHC-32 | Dawan | 75.00 | 1.0 | 0.05 | 19.57 |  |  |  |  |  |  |
| HHC-31 | Dawan | 73.00 | 0.5 | 0.03 | 19.92 |  |  |  |  |  |  |
| HHC-30 | Dawan | 71.00 | 0.5 | 0.04 | 12.14 |  |  |  |  |  |  |
| HHC-29 | Dawan | 69.00 | 1.1 | 0.03 | 34.06 |  |  |  |  |  |  |
| HHC-28 | Dawan | 67.00 | 0.9 | 0.03 | 27.95 |  |  |  |  |  |  |
| HHC-27 | Dawan | 65.00 | 0.7 | 0.02 | 32.41 |  |  |  |  |  |  |
| HHC-26 | Dawan | 63.00 | 0.8 | 0.03 | 29.63 |  |  |  |  |  |  |
| HHC-25 | Dawan | 59.00 | 1.6 | 0.02 | 64.52 |  |  |  |  |  |  |
| HHC-24 | Dawan | 57.00 | 2.2 | 0.10 | 21.93 |  |  |  |  |  |  |
| HHC-23 | Dawan | 55.00 | 1.2 | 0.09 | 12.89 |  |  |  |  |  |  |
| HHC-22 | Dawan | 53.00 | 1.9 | 0.03 | 61.29 |  |  |  |  |  |  |
| HHC-21 | Dawan | 51.00 | 2.3 | 0.03 | 66.86 |  |  |  |  |  |  |
| HHC-20 | Dawan | 49.00 | 1.5 | 0.03 | 47.62 |  |  |  |  |  |  |
| HHC-19 | Dawan | 47.00 | 2.4 | 0.03 | 91.95 |  |  |  |  |  |  |
| HHC-18 | Dawan | 45.00 | 2.1 | 0.02 | 98.13 |  |  |  |  |  |  |
| HHC-17 | Dawan | 43.00 | 0.8 | 0.03 | 24.92 |  |  |  |  |  |  |
| HHC-16 | Dawan | 41.00 | 2.0 | 0.03 | 77.22 |  |  |  |  |  |  |
| HHC-15 | Dawan | 39.00 | 1.6 | 0.04 | 42.90 |  |  |  |  |  |  |
| HHC-14 | Dawan | 37.00 | 1.3 | 0.03 | 44.52 |  |  |  |  |  |  |
| HHC-13 | Dawan | 35.00 | 3.0 | 0.04 | 79.16 |  |  |  |  |  |  |
| HHC-12 | Dawan | 33.00 | 2.8 | 0.03 | 90.61 |  |  |  |  |  |  |
| HHC-11 | Dawan | 31.00 | 3.6 | 0.06 | 61.33 |  |  |  |  |  |  |
| HHC-10 | Dawan | 29.00 | 1.6 | 0.02 | 98.77 |  |  |  |  |  |  |
| HHC-9 | Dawan | 27.00 | 1.2 | 0.02 | 48.19 |  |  |  |  |  |  |
| HHC-8 | Hunghuayuan | 24.00 | 3.1 | 0.11 | 27.41 |  |  |  |  |  |  |
| HHC-7 | Hunghuayuan | 21.00 | 1.9 | 0.03 | 59.19 |  |  |  |  |  |  |
| HHC-6 | Hunghuayuan | 18.00 | 2.5 | 0.14 | 17.59 |  |  |  |  |  |  |
| HHC-5 | Hunghuayuan | 15.00 | 2.3 | 0.11 | 20.16 |  |  |  |  |  |  |
| HHC-4 | Hunghuayuan | 12.00 | 2.1 | 0.02 | 85.37 |  |  |  |  |  |  |
| HHC-3 | Hunghuayuan | 9.00 | 4.2 | 0.11 | 37.17 |  |  |  |  |  |  |
| HHC-2 | Hunghuayuan | 6.00 | 2.0 | 0.16 | 12.45 |  |  |  |  |  |  |
| HHC-1 | Hunghuayuan | 3.00 | 3.3 | 0.16 | 20.57 |  |  |  |  |  |  |

**Supplementary Table S2.** Hg concentrations, TOC values, Mo values and Hg isotopic compsition for Dingjiapo samples. These data are plotted in Figure 3 and were used to select a representative subset of samples for Hg isotope analyses in this study.

| Sample No. | Formation | Height (m) | Hg (ppb) | TOC (%) | Hg/TOC (ppb/%) | Mo (ppm) | Hg/Mo (‰) | δ^202^Hg (‰) | 2σ | Δ^199^Hg (‰) | 2σ |
| --- | --- | --- | --- | --- | --- | --- | --- | --- | --- | --- | --- |
| DJP-53 | Lungmachi | 4.34 | 180.2 | 1.62 | 110.92 |  |  |  |  |  |  |
| DJP-52 | Lungmachi | 4.28 | 180.6 | 2.02 | 89.52 |  |  |  |  |  |  |
| DJP-51 | Lungmachi | 4.22 | 83.5 | 2.50 | 33.39 | 15.53 | 5.38 | -0.48 | 0.07 | 0.11 | 0.04 |
| DJP-50 | Lungmachi | 4.16 | 71.2 | 1.63 | 43.69 |  |  |  |  |  |  |
| DJP-49 | Lungmachi | 4.10 | 111.7 | 1.27 | 87.96 |  |  |  |  |  |  |
| DJP-48 | Lungmachi | 4.04 | 95.3 | 1.70 | 56.12 |  |  |  |  |  |  |
| DJP-47 | Lungmachi | 3.98 | 101.2 | 1.42 | 71.07 | 21.39 | 4.73 | -1.05 | 0.05 | 0.18 | 0.01 |
| DJP-46 | Lungmachi | 3.92 | 146.3 | 2.15 | 68.01 |  |  |  |  |  |  |
| DJP-45 | Lungmachi | 3.86 | 246.1 | 2.42 | 101.57 |  |  |  |  |  |  |
| DJP-44 | Lungmachi | 3.80 | 134.3 | 1.10 | 122.47 |  |  |  |  |  |  |
| DJP-43 | Lungmachi | 3.74 | 100.5 | 1.43 | 70.21 | 25.95 | 3.87 | -1.62 | 0.05 | 0.14 | 0.01 |
| DJP-42 | Lungmachi | 3.68 | 83.2 | 2.84 | 29.33 |  |  |  |  |  |  |
| DJP-41 | Lungmachi | 3.62 | 159.7 | 1.31 | 122.08 | 34.34 | 4.65 | -0.83 | 0.03 | 0.16 | 0.01 |
| DJP-40 | Lungmachi | 3.56 | 105.2 | 0.97 | 108.51 |  |  |  |  |  |  |
| DJP-39 | Lungmachi | 3.50 | 180.9 | 0.84 | 214.26 | 19.42 | 9.32 | -1.22 | 0.07 | 0.10 | 0.03 |
| DJP-38 | Lungmachi | 3.44 | 87.4 | 0.91 | 95.81 |  |  |  |  |  |  |
| DJP-37 | Lungmachi | 3.38 | 198.9 | 1.08 | 183.67 |  |  |  |  |  |  |
| DJP-36 | Lungmachi | 3.32 | 225.6 | 1.31 | 171.95 |  |  |  |  |  |  |
| DJP-35 | Lungmachi | 3.26 | 164.1 | 1.42 | 115.68 |  |  |  |  |  |  |
| DJP-34 | Lungmachi | 3.20 | 39.6 | 0.22 | 182.72 | 6.86 | 5.77 | -1.26 | 0.04 | 0.15 | 0.01 |
| DJP-33 | Lungmachi | 3.14 | 56.2 | 0.62 | 90.71 |  |  |  |  |  |  |
| DJP-32 | Lungmachi | 3.08 | 138.9 | 0.78 | 177.18 |  |  |  |  |  |  |
| DJP-31 | Kuanyinchiao | 3.02 | 187.3 | 0.72 | 259.50 | 111.40 | 1.68 | -1.21 | 0.02 | 0.13 | 0.00 |
| DJP-30 | Kuanyinchiao | 2.94 | 203.7 | 0.76 | 268.94 | 26.59 | 7.66 | -0.54 | 0.04 | 0.13 | 0.01 |
| DJP-29 | Kuanyinchiao | 2.86 | 105.5 | 0.68 | 154.71 |  |  |  |  |  |  |
| DJP-28 | Kuanyinchiao | 2.78 | 101.3 | 0.59 | 170.86 | 5.13 | 19.73 | -1.05 | 0.03 | 0.16 | 0.01 |
| DJP-27 | Wufeng | 2.70 | 60.1 | 0.64 | 93.85 |  |  |  |  |  |  |
| DJP-26 | Wufeng | 2.60 | 61.3 | 0.81 | 75.35 |  |  |  |  |  |  |
| DJP-25 | Wufeng | 2.50 | 35.3 | 0.84 | 41.90 | 6.42 | 5.49 | -1.20 | 0.00 | 0.24 | 0.01 |
| DJP-24 | Wufeng | 2.40 | 33.2 | 1.03 | 32.39 |  |  |  |  |  |  |
| DJP-23 | Wufeng | 2.30 | 88.5 | 0.80 | 110.72 |  |  |  |  |  |  |
| DJP-22 | Wufeng | 2.20 | 318.0 | 0.93 | 342.50 | 83.85 | 3.79 | -0.44 | 0.04 | 0.11 | 0.01 |
| DJP-21 | Wufeng | 2.10 | 124.6 | 1.10 | 113.17 |  |  |  |  |  |  |
| DJP-20 | Wufeng | 2.00 | 406.1 | 1.34 | 302.37 | 72.24 | 5.62 | -0.58 | 0.01 | 0.05 | 0.01 |
| DJP-19 | Wufeng | 1.90 | 295.7 | 1.00 | 295.53 | 86.15 | 3.43 | -0.40 | 0.00 | 0.08 | 0.00 |
| DJP-18 | Wufeng | 1.80 | 27.7 | 1.40 | 19.77 |  |  |  |  |  |  |
| DJP-17 | Wufeng | 1.70 | 30.8 | 1.15 | 26.90 |  |  |  |  |  |  |
| DJP-16 | Wufeng | 1.60 | 105.9 | 1.48 | 71.64 | 31.91 | 3.32 | -0.60 | 0.07 | 0.14 | 0.04 |
| DJP-15 | Wufeng | 1.50 | 40.8 | 2.05 | 19.93 |  |  |  |  |  |  |
| DJP-14 | Wufeng | 1.40 | 106.4 | 1.43 | 74.62 |  |  |  |  |  |  |
| DJP-13 | Wufeng | 1.30 | 109.8 | 1.30 | 84.18 |  |  |  |  |  |  |
| DJP-12 | Wufeng | 1.20 | 28.6 | 0.95 | 30.26 | 25.13 | 1.14 | -0.95 | 0.04 | 0.12 | 0.01 |
| DJP-11 | Wufeng | 1.10 | 25.6 | 0.92 | 27.79 |  |  |  |  |  |  |
| DJP-10 | Wufeng | 1.00 | 62.7 | 2.52 | 24.87 |  |  |  |  |  |  |
| DJP-9 | Wufeng | 0.90 | 55.2 | 2.05 | 26.89 |  |  |  |  |  |  |
| DJP-8 | Wufeng | 0.80 | 113.9 | 1.59 | 71.76 |  |  |  |  |  |  |
| DJP-7 | Wufeng | 0.70 | 103.1 | 1.60 | 64.31 | 31.66 | 3.26 | -0.90 | 0.00 | 0.11 | 0.00 |
| DJP-6 | Wufeng | 0.60 | 34.3 | 1.32 | 26.03 |  |  |  |  |  |  |
| DJP-5 | Wufeng | 0.50 | 62.6 | 2.48 | 25.22 |  |  |  |  |  |  |
| DJP-4 | Wufeng | 0.40 | 37.7 | 1.48 | 25.48 |  |  |  |  |  |  |
| DJP-3 | Wufeng | 0.30 | 44.3 | 1.46 | 30.30 | 27.87 | 1.59 | -0.89 | 0.02 | 0.17 | 0.01 |
| DJP-2 | Wufeng | 0.20 | 25.3 | 1.30 | 19.52 |  |  |  |  |  |  |
| DJP-1 | Wufeng | 0.10 | 51.9 | 1.93 | 26.84 |  |  |  |  |  |  |
